# Metabolism before, during and after anaesthesia in colic and healthy horses

**DOI:** 10.1186/1751-0147-49-34

**Published:** 2007-11-15

**Authors:** Anna H Edner, Görel C Nyman, Birgitta Essén-Gustavsson

**Affiliations:** 1 Department of Clinical Sciences, Faculty of Veterinary Medicine and Animal Science, Swedish University of Agricultural Sciences, Uppsala, Sweden; 2 Department of Medical Sciences, Clinical Physiology, University hospital, Uppsala, Sweden

## Abstract

**Background:**

Many colic horses are compromised due to the disease state and from hours of starvation and sometimes long trailer rides. This could influence their muscle energy reserves and affect the horses' ability to recover. The principal aim was to follow metabolic parameter before, during, and up to 7 days after anaesthesia in healthy horses and in horses undergoing abdominal surgery due to colic.

**Methods:**

20 healthy horses given anaesthesia alone and 20 colic horses subjected to emergency abdominal surgery were anaesthetised for a mean of 228 minutes and 183 minutes respectively. Blood for analysis of haematology, electrolytes, cortisol, creatine kinase (CK), free fatty acids (FFA), glycerol, glucose and lactate was sampled before, during, and up to 7 days after anaesthesia. Arterial and venous blood gases were obtained before, during and up to 8 hours after recovery. Gluteal muscle biopsy specimens for biochemical analysis of muscle metabolites were obtained at start and end of anaesthesia and 1 h and 1 day after recovery.

**Results:**

Plasma cortisol, FFA, glycerol, glucose, lactate and CK were elevated and serum phosphate and potassium were lower in colic horses before anaesthesia. Muscle adenosine triphosphate (ATP) content was low in several colic horses. Anaesthesia and surgery resulted in a decrease in plasma FFA and glycerol in colic horses whereas levels increased in healthy horses. During anaesthesia muscle and plasma lactate and plasma phosphate increased in both groups. In the colic horses plasma lactate increased further after recovery. Plasma FFA and glycerol increased 8 h after standing in the colic horses. In both groups, plasma concentrations of CK increased and serum phosphate decreased post-anaesthesia. On Day 7 most parameters were not different between groups. Colic horses lost on average 8% of their initial weight. Eleven colic horses completed the study.

**Conclusion:**

Colic horses entered anaesthesia with altered metabolism and in a negative oxygen balance. Muscle oxygenation was insufficient during anaesthesia in both groups, although to a lesser extent in the healthy horses. The post-anaesthetic period was associated with increased lipolysis and weight loss in the colic horses, indicating a negative energy balance during the first week post-operatively.

## Background

An approximately ten-fold higher incidence of anaesthetic-related deaths has been reported in colic horses undergoing emergency abdominal surgery in comparison with healthy horses undergoing elective anaesthetic procedures [1-3]. Attempts have been made in several studies to identify parameters that may be used to predict the probability of survival in colic horses [4-12]. The best predictors seem to be parameters that assess the cardiovascular function of the horse. The progress of different clinical-chemical parameters has been studied in venous or arterial blood during and after anaesthesia in horses subjected to emergency abdominal surgery [9,13-15]. Metabolic changes that occur locally in a muscle can be studied by analysis of muscle biopsy specimens and microdialysis techniques. Studies have shown that anaesthesia in healthy horses is associated with anaerobic metabolism observed as a degradation of adenosine triphosphate (ATP) and creatine phosphate (CP) and production of lactate within the muscle [16,17]. This may be related to general hypoperfusion caused by the anaesthetic agents per se [18] or to compressive forces, or both restricting local blood perfusion [19,20].

In the colic horse, the normal metabolic rate and pathways are altered by several factors such as circulatory insufficiency, endotoxaemia and acid-base disorders. In addition, the horses are in pain, have starved for hours or up to several days, and often have been transported for some distance. All these factors are potential sources of stress that result in an increased demand for energy.

We hypothesised that colic horses enter anaesthesia in a state of metabolic stress causing muscle metabolic changes that postoperatively differ from that in healthy horses recovering from anaesthesia. The aim of this study was therefore to follow metabolic parameters in colic horses and in healthy horses by analysing blood and muscle biopsy samples before, during, and up to 7 days after anaesthesia.

## Methods

### Study design

This was a prospective clinical study performed on colic horses with a reference group consisting of clinically healthy research horses submitted to an experimental procedure. The study was approved by the Ethical Committee on Animal Experiments in Uppsala, Sweden.

### Colic horses

The study comprised 20 horses subjected to acute abdominal surgery (referred to as C1–C20) at the horse clinic of the Swedish University of Agricultural Sciences (SLU), from January to April 2001 and from January to June 2002. Information regarding breed, age, sex, weight and total duration of colic is given in Tables 1 and 2. The horses were referred by field practitioners or smaller equine clinics because of unresolved acute colic of varying aetiology. The mean (± SD) distance travelled was 103 (83) km. All colic horses but two had been treated immediately before referral, in most cases with an analgesic or spasmolytic drug (dipyrone, detomidine, butorphanol, flunixin meglumin). Other administered drugs were intravenous vitamin B, antibiotics, orally administered mineral oil and water, and intravenous (IV) electrolytes.

**Table 1:** Description of the 20 colic and 20 healthy horses included in the study

	**Colic horses**	**Healthy horses**
**Weight**	527 ± 106 kg (230–698)	495 ± 47 kg (411–584)
**Age**	11 ± 6 years (2–22)	8 ± 5 years (3–19)
**Sex**	10 mares, 8 geldings, 2 stallions	12 mares, 8 geldings

For weight and age the mean values (± SD) are given with the range within parentheses.

**Table 2:** Anaesthesia, recovery and survival rates in 20 colic and 20 healthy horses

	**Colic horses**	**Healthy horses**
**Duration of anaesthesia**	183 ± 62 mins (45–300)	228 ± 26 mins (189–273)
**Recovery time to standing**	64 ± 45 mins (15–180)	52 ± 19 mins (18–86)
**Number of attempts to stand**	1.4 ± 0.6 (1–3)	2.4 ± 1.2 (1–4)
**Survival rate**	11/20	20/20
**Time of death/euthanasia**	3 during anaesthesia, 2 in recovery, 2 within 24 h after standing, 2 between DAY 2–DAY 7	
**Reasons for euthanasia or death**	2 circulatory failure, 1 acute myocardial degeneration (autopsy diagnosis), 2 surgical findings, 1 ruptured stomach, 1 laminitis, 1 endotoxaemia, 1 endocarditis (chronic but not diagnosed before anaesthesia)	

The mean values (± SD) are given with the range within parentheses.

EHV = equine herpes virus; DAY 2 and 7 = 2 and 7 days after anaesthesia.

On arrival at SLU, all horses were examined clinically by the veterinarian on duty. Therapy was initiated immediately according to the severity of the clinical signs and the clinic routines and consisted of administration of an analgesic or spasmolytic agent as stated above or of xylazine, romifidine and hyoscine butylbromide, administration of intravenous electrolytes (Ringer acetat, Pharmacia & Upjohn, Sweden) and/or a dextran colloid (Macrodex^®^, Meda AB, Solna, Sweden). Other treatment before surgery consisted of antibiotics and a booster dose of tetanus vaccine. The decision concerning surgery was taken by the clinician. The approximate length of time (and duration of food withdrawal) from the observation of colic signs to the time of surgery varied from 3 hours up to 2.5 days, with a median of 14 hours. All colic horses destined for acute abdominal surgery whose owner gave their informed consent to participation entered the study. The study was closed when 20 horses (5 Standardbred trotters, 10 Warmblooded riding horses, 1 Shetland pony, 1 Welsh cob, 1 pony cross, 1 Arabian and 1 Icelandic horse) had entered.

When the preoperative clinical status was judged retrospectively, four colic horses were considered to have been in a markedly worse condition than the other colic horses, and these four are referred to as ASA 5 (American Society of Anaesthesiologists physical status grade 5). The other colic horses were regarded as ASA 4.

### Reference horses

As a reference group, 20 healthy, Standardbred trotters (referred to as H1–H20) owned by the former Department of Large Animal Clinical Sciences, SLU, Uppsala, Sweden, were studied. They are hereafter referred to as the "healthy horses". These horses were anaesthetised in dorsal recumbency for participation in two other anaesthesia research projects and data was collected in January 2000 and October 2001 (Table 2). In these horses the effect on peripheral perfusion was studied during spontaneous breathing and/or mechanical ventilation with intermittent positive pressure ventilation. Prior to the study no horse had shown clinical signs of disease or was receiving any treatment, and none had a recent history of colic. They were housed at the department, where they were kept outdoor during the day and stabled at night. They were fasted for 12 hours before anaesthesia.

### Anaesthesia

#### Colic horses

Since the colic horses had been medicated by the referral veterinarian and by the clinician at the University clinic, additional premedication with low a dose of an alpha-2 adrenoceptor agonist and butorphanol was only given to a few horses before induction. In 15 horses, anaesthesia was induced with an intravenous infusion of guaifenesin (Myolaxin^®^vet, diluted to 7.5%, Vétoquinol AG, Belp, Switzerland) to effect and a bolus dose of 3.1–4.4 mg/kg thiopentone sodium (Pentothal^®^Natrium, Electra-Box Pharma AB, Tyresö, Sweden). Ketamine (1.9–2.4 mg/kg IV, Ketaminol^®^vet, Intervet AB, Danderyd, Sweden) with diazepam (0.02–0.03 mg/kg IV, Diazepam-ratiopharm 10, PharmaMedics, Bassersdorf, Switzerland) was used for induction in three horses. In two horses anaesthesia was induced with guaifenesin and ketamine (1.6 mg/kg and 2.1 mg/kg IV respectively). After intubation, the horses were transported into the theatre and placed in dorsal recumbency on a medical foam mattress (Tempur AB, DanFoam, Denmark) with the hind limbs supported in a semi-flexed position. In all horses, anaesthesia was maintained with isoflurane in oxygen delivered by a semi-closed large animal anaesthetic circuit. Breathing was spontaneous during the whole anaesthetic procedure in 13 horses and was controlled using intermittent positive pressure ventilation (IPPV) for most or part of the procedure in 7 horses. During anaesthesia, all horses were given an IV infusion of Ringer acetate. To keep the mean systemic arterial blood pressure (MSAP) above 70 mmHg, a dextran colloid, up to 10 mL/kg, was administered IV. If no effect was seen within 30 minutes or if mean systemic arterial pressure (MSAP) was below 50 mmHg, dobutamine was given symptomatically IV (0.5–5 μg/kg/min) to maintain or reach an MSAP of 70 mmHg. After anaesthesia the horses were allowed to recover in a padded stall before being taken to their stables. The horses were extubated after the swallowing reflex had returned or when in sternal recumbency if gastric regurgitation was suspected. Oxygen was insufflated at 15 L/min through a nostril until the horse gained the sternal position.

#### Healthy horses

The healthy horses were premedicated with detomidine (10 μg/kg IV, Domosedan vet, Orion, Animal Health, Sollentuna, Sweden) and 10 minutes later anaesthesia was induced IV with guaifenesin to effect and a bolus dose of thiopentone sodium (4.5 mg/kg IV). Intubation and maintenance of anaesthesia were as described above. In ten horses IPPV was used during the whole procedure and 9 horses were ventilated both by spontaneous breathing and IPPV. One horse was breathing spontaneously for the whole procedure. During anaesthesia, all horses were given an infusion of Ringer acetate. After anaesthesia the horses were allowed to recover in a padded stall as described above. Fourteen of the 20 healthy horses were given xylazine and flunixin after discontinuation of inhalation anaesthesia. No recovery assistance was given.

### Post anaesthesia

Medical treatment in the 7-day observation period after anaesthesia was provided by the treating veterinarian as judged by the horse's condition.

Feed was provided to the colic horses at the decision of the clinician in charge and consisted of increasing rations of hay and a wet mixture of beet pulp, wheat and barley bran. The horses were hand-walked several times daily. The healthy horses were provided with water and hay (approximately 8 kg/day) when they had fully recovered from anaesthesia, and were turned out into a paddock the day after anaesthesia. They were fed the wet mixture described for the colic horses at 0.5–1 kg/day.

### Haemodynamic, respiratory, and blood gas measurements

During anaesthesia MSAP, heart rate (HR), oxygen saturation and an electrocardiogram (ECG) were monitored (Datex light, Datex Engström Instrumentation Corporation, Helsinki, Finland). Blood pressure was measured invasively through a catheter in a facial artery. In two cases where a permanent catheter failed to function, arterial blood pressure was measured non-invasively (oscillometrically) with a pneumatic cuff placed around the tail base. Respiratory parameters (expired volume, inhaled and exhaled isoflurane, carbon dioxide and oxygen and in the case of IPPV, peak inspiratory pressure and expiratory volumes) were monitored by side-stream spirometry (Capnomac Ultima, Datex Engström Instrumentation Corporation, Helsinki, Finland). Respiratory rate was counted by observing the costo-abdominal movements. Physiological parameters were assessed before anaesthesia (HR, RR, mucous membranes; MM, capillary refill time; CRT, peripheral pulse) and in 5-minute intervals during anaesthesia (HR, RR, MM, CRT, peripheral pulse). Until the standing position was reached, the horses were examined every 10–30 minutes and after recovery at least every hour during the first 24 hours (HR, RR, mucous membranes, peripheral pulse).

Arterial (a) and jugular venous (v) blood samples were drawn into heparinised syringes, placed on ice and analysed within 10 minutes for oxygen and carbon dioxide tensions (PO_2_, PCO_2_), pH and haemoglobin saturation of oxygen (SatO_2_) while bicarbonate (HCO_3_^-^) and base excess (BE) were calculated (ABL™5, Radiometer Medical A/S, Copenhagen, Denmark). A correction for current rectal temperature was made. Blood gases (a, v) were obtained immediately after induction and every hour during anaesthesia in all horses. Before anaesthesia venous blood gas samples were obtained from six colic horses and two healthy horses. After anaesthesia and up to eight hours after recovery to standing venous blood gas samples were obtained from eight colic and seven healthy horses.

### Samples

#### Sampling and analyses of blood

Venous blood was sampled in the awake state before induction (PRE), at every hour of anaesthesia (AN 1, AN 2, etc), 15 minutes and every hour after discontinuation of inhalation anaesthesia while the horse was still recumbent (REC 15', REC 1, etc), 15 and 30 minutes, and 1, 2, 4, 8, 12, and 24 hours after standing (POST 15', POST 30', POST 1, POST 2, etc and DAY 1), and thereafter at 24-hour intervals for 7 days after anaesthesia (DAY 2, DAY 3, etc).

The blood samples were collected from a catheter in the jugular vein. Samples for assays of plasma lactate, glycerol, glucose, free fatty acids (FFA), cortisol and creatine kinase (CK) were taken in heparinised vials, while vials containing no additive were used for measurements of serum sodium, potassium, chloride, total calcium and inorganic phosphate total protein and albumin. Samples were kept on ice until they were centrifuged (within 30 minutes) and stored at -80°C until analysed. Blood for determination of haemoglobin (Hb), haematocrit (Hct) and white blood cell count (WBC) was collected in EDTA vials, and stored at 5°C until analysed within 36 hours.

The plasma lactate concentration was assayed with a lactate analyser (Analox GM7, Analox Ltd, London, Great Britain). Glycerol was determined using a commercial kit (EnzyPlus, Diffchamb AB, Västra Frölunda, Sweden). Glucose and CK were assayed by modified fluorometric methods [21]. FFA was determined with a kit from Wako (NEFA C test, Wako Chemicals GmbH, Neuss, Germany). Plasma cortisol was measured by a competitive immunoassay method (Immulite Cortisol, DPC, Los Angeles, CA, USA). Serum sodium, potassium chloride, total calcium, inorganic phosphate and albumin concentrations were determined by a spectrophotometric method using standardized reagent kits (Konelab 30, Kone Instruments, Espoo, Finland). Total protein was determined by refractometry. Hb was measured with a quantitative reflectance test (Reflotron, Boeringer Mannheim Scandinavia AB, Bromma, Sweden), and for Hct measurement a capillary microcentrifuge was used. The total and differential WBC were determined by a spectrophotometric test (CELL-DYN 3500, ABBOTT, Abbott Laboratories, Abbott Park, IL, USA).

#### Muscle biopsy sampling and analyses

A biopsy specimen was obtained from the right gluteus medius immediately after induction of anaesthesia (AN START) and at the end of anaesthesia (AN END) in all horses. In six colic horses and in seven healthy horses a sample was obtained 1 hour after recovery to standing (POST 1). In 13 colic horses and in the seven healthy horses, a biopsy sample was also obtained the day after surgery (DAY 1).

The muscle samples were taken from a site half-way on a midline between the distal border of the tuber coxae and the tail base. Samples were obtained with a Bergström muscle biopsy needle (external diameter 5 mm) after surgical preparation and, in the awake horse, after local analgesia, 2 mL of 2% lidocaine (Xylocain, AstraZeneca AB, Södertälje, Sweden) instilled subcutaneously and under the fascia. A 10-mm incision was made through the skin and fascia with a scalpel and muscle samples were obtained from a site 5–6 cm deep into the muscle belly. Subsequent biopsy samples were obtained through the same incision. The samples were immediately frozen in liquid nitrogen and stored at -80° until analysed. They were freeze-dried, dissected free from connective tissue, blood and fat, and then weighed (1–2 mg dry weight; d.w.) and extracted in perchloric acid before being neutralized with potassium hydroxide.

The concentrations of adenine nucleotides (ATP, adenosine diphosphate; ADP, adenosine monophosphate AMP) and inosine monophosphate (IMP) were determined by a modified high performance liquid chromatography (HPLC) technique using a C:18 (250 × 4.6, 5 mm) column [22]. CP and creatine were determined with an HPLC technique [23]. Muscle lactate was assayed by a modified fluorometric method [21].

### Other measurements and observations

All horses were weighed before anaesthesia. The colic horses and seven healthy horses were weighed after recovery, before being taken to their stables and, when possible, daily until DAY 7. The same scales were used at all time points. Unfortunately, these were not calibrated between each horse. Rectal temperature was measured in all horses before, at every hour and at the end of anaesthesia. Thereafter rectal temperature was measured immediately before each sampling for measurement of blood gases. The gait and movements at walk were examined after recovery and daily if any signs of lameness or limb dysfunction were seen at recovery. Any other occurring complications such as diarrhoea and laminitis were noted.

### Statistical analysis

Comparisons of plasma samples concentrations between groups at PRE were performed using Mann-Whitney U-test for variables not being normally distributed and Student's t-test for independent samples for those variables with normal distribution (Statistica 6.0 and 7.0, StatSoft^®^, Inc. Tulsa OK, USA).

Changes from PRE to END for blood analytes, HR, MSAP and temperature, and from PRE to POST 4 for pHv were analysed with an ANOVA for repeated measures followed by Tukey Post Hoc test for unequal N or planned comparisons when the sphericity assumptions were violated. If the interaction Group*Time was significant, simple effects were examined, i.e. effects of one factor holding the other factor fixed. The p-values were then corrected according to the Bonferroni procedure. When Levene's test for homogeneity of variances was significant, an ANOVA model with separate variance estimates was used, Proc Mixed in SAS (SAS^® ^System 9.1, SAS Institute Inc., Cary, NC, USA). In these analyses, a p-value of < 0.05 was considered significant. Mixed model repeated measures analyses (Proc Mixed in SAS) were used to examine the pattern of change in the blood variables from PRE to postoperative period up to one week after anaesthesia. Different covariance pattern models were tested, compound symmetry, heterogeneous compound symmetry, first order autoregressive and heterogeneous first order autoregressive models. When the variances in the two groups were inhomogeneous, separate covariance pattern was estimated for each group. The covariance structure with the smallest value of Akaike's Information Criterion was considered most appropriate. Group and Time were modelled as fix factors. The Group*Time interaction refers to the statistical test of whether the mean change over time is the same for the two groups. In case of a significant interaction, simple effects were examined, i.e. effects of one factor holding the other factor fixed. The distribution of CK, glycerol and lactate were positively skewed and were log transformed before formal analyses. Due to multiple comparisons, significance was considered when p < 0.01 [24-26].

Changes in weight and the results from muscle biopsy sample assays were analysed with a Mann-Whitney U test for comparisons between groups and a Friedman ANOVA for analysis of changes within groups (Statistica 6.0). P < 0.05 was considered statistically significant.

Samples from the colic horses with the poorest prognosis of survival (C2, C8, C10, C14), as judged from their pre-operative status and findings during surgery, were not included in the statistical analyses and are also shown separately in the tables and figures. Results are given as the mean value and the standard deviations.

## Results

### Outcome

Of the 20 colic horses entering the study, 11 horses completed the study, i.e., were alive one week after surgery and subsequently discharged from the hospital. Surgical diagnoses were; 2 colon impactions, 8 colon displacements, 1 colon necrosis, 1 small intestine volvolus, 2 small intestine incarcerations, 4 strangulations, 3 enteritis/colitis, 2 peritonitis and 1 abdominal neoplasia.

All colic horses that recovered from anaesthesia were given IV fluids, antibiotics (penicillin and/or gentamicin) and flunixin. After recovery from anaesthesia complications developed in 11 colic horses; 3 diarrhoea, 3 peritonitis, 5 toxinaemia, 1 hypocalcaemia, 1 aspiration pneumonia and 1 laminitis. Eight colic horses developed some type of gait disturbance post anaesthesia but only in four horses a clinical diagnosis of post-anaesthetic myositis with swollen, painful muscles was made. In these horses symptoms disappeared within five days. In the other four horses, no definite diagnosis could be made but the horses were walking normally within 12–24 hours.

Specific treatment was required in a total of eight horses, and apart from additional analgesia (as stated above but could also include pethidine and IV infusions of lidocaine), and electrolyte infusions this treatment included IV infusions of potassium plus 2.5% glucose in two horses (newly foaled, inappetent mares), glucose only in one horse (hyperlipaemia prophylaxis), and a calcium infusion in one horse (hypocalcaemia).

All healthy horses completed the study period. Complications in the post-anaesthetic period occurred in seven healthy horses. Four horses showed some degree of gait dysfunction but only two had palpably sore muscles. One of these horses developed a severe triceps myopathy (H14) and was also treated with flumethazone and topical ketoprofen gel, while the other horse only showed mild symptoms. The two other horses were walking normally within 12–24 hours. Three horses developed fever and were treated with penicillin. Equine herpes virus infection was diagnosed in two of these horses (the third horse was not sampled). Two horses showed slight colic symptoms within the first 24 hours after recovery and were treated symptomatically (IV fluids, flunixin, dipyrone).

Information regarding anaesthesia time, recovery, reasons for death or euthanasia and when available, the post mortem diagnosis, in colic and healthy horses are given in Table 2.

### Hemodynamics, blood gas measurements and anaesthesia

During anaesthesia the temperature decreased steadily from 37.9 ± 0.8°C in the ASA 4 colic horses and 37.5 ± 0.3°C in the healthy horses before anaesthesia, to 35.4 ± 1.3°C and 34.3 ± 1.0°C in colic and healthy horses respectively at REC 15', after which temperatures increased. Heart rate was higher in colic than in healthy horses at PRE (55 ± 11 and 36 ± 4 beats/min respectively; p = 0.0003) and at the end of anaesthesia (47 ± 12 and 34 ± 4 beats/min respectively; p = 0.009). At PRE the HR ranged from 36–80 beats/min in the ASA 4 horses and from 60–80 beats/min in the ASA 5 horses. There were no statistically significant differences in MSAP between colic (68 ± 25 and 75 ± 12 mmHg at AN 1 and END respectively) and healthy horses (73 ± 11 and 86 ± 13 mmHg) during anaesthesia. MSAP was below 50 mmHg during some period in five colic horses and in one healthy horse. The lowest MSAP in a surviving colic horse was 25 mmHg. Apart from electrolyte infusions, additional treatment for hypotension was provided during anaesthesia in 17 colic horses. Seven healthy horses received treatment with dobutamine to keep MSAP stable between 70 and 90 mmHg. No treatment was given to three healthy horses despite periodic hypotension due to the research protocol in these horses. The total infusion rate of fluids during anaesthesia in the colic horses was 10 mL/kg/h and in the healthy horses 4 mL/kg/h.

The pH in arterial blood during anaesthesia was significantly lower (p < 0.0001) in colic horses (7.24 ± 0.09) than in healthy horses (7.44 ± 0.05). The lowest measured arterial pH during anaesthesia was 6.97 in a surviving colic horse (C1) and this was due to a combined respiratory and metabolic (BE: -14) acidosis. In six colic horses from which a preoperative venous blood gas sample was obtained, the venous pH varied between 7.20 and 7.39 and BE varied between 5 and -12. Venous pH was lower in colic horses until POST 30'. PaO_2 _was below 8.0 kPa in five of the colic horses (of which only one was ASA 5) and in eight of the healthy horses during some part of the anaesthetic procedure. One of the surviving colic horses (C5) never achieved a higher PaO_2 _than 4.5 kPa.

The colic horses were anaesthetised for 183 (62) minutes (range 45–300 minutes) and the healthy horses for 228 (26) minutes (range 189–273 minutes). The mean end tidal isoflurane was 1.5% (0.5) in the colic and 1.6% (0.2) in the healthy horses.

### Haematology

**Figure 1:**
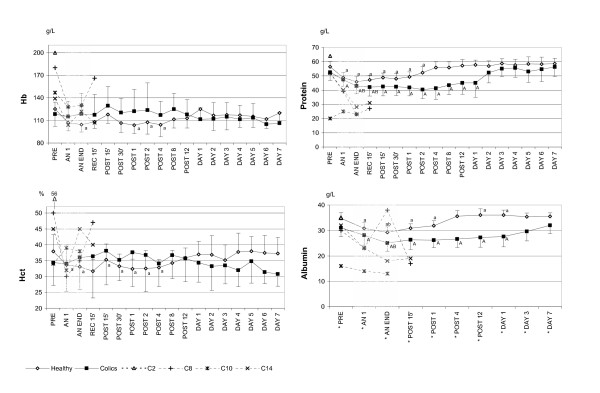
**Concentrations of haemoglobin, hematocrit, serum protein and albumin in healthy and colic horses**. The mean values (SD) are shown from before anaesthesia to 7 days post anaesthesia in healthy (n = 11–20) and colic horses (n = 10–16) with the American Society of Anaesthesiologists physical status 5 (ASA 5) colic horses shown individually. Note that the time scale is not linear. Hb = haemoglobin; Hct = haematocrit; PRE = before anaesthesia; AN 1 = after one hour of anaesthesia; AN END = end of anaesthesia; REC 15' = 15 minutes after discontinuation of inhalation anaesthesia, still recumbent; POST 15' and 30' = 15 and 30 minutes after recovery to standing; POST 1, 2, 4, 8, 12 = hours after standing; DAY 1, 2, 3, 4, 5, 6, 7 = days after anaesthesia. * Significant difference (p < 0.05) between groups. ^A ^(colics)^a ^(healthy) = significantly different from PRE. ^B ^(colics)^b ^(healthy) = significantly different from AN 1.

Changes in Hct and Hb from PRE to DAY 7 are shown in Figure 1.

White blood cell count (× 10^9^/L) did not differ between the groups at PRE (6.1 ± 2.7 in colic horses and 6.5 ± 1.2 in healthy horses), but the range was wide in the colic horses (2.3–11.0). From PRE to DAY 1 there was a decrease (p = 0.04) in WBC in the colic horses (3.7 ± 1.7), but an increase (p < 0.0001) in the healthy horses (8.6 ± 1.5). On DAY 7 WBC in colic horses had increased (p = 0.002) to 9.5 (2.4), while no further change was seen in the healthy horses. The ASA 5 horses did not differ in WBC from other colic horses (range; 2.2–10.8).

### Blood chemistry

**Figure 2:**
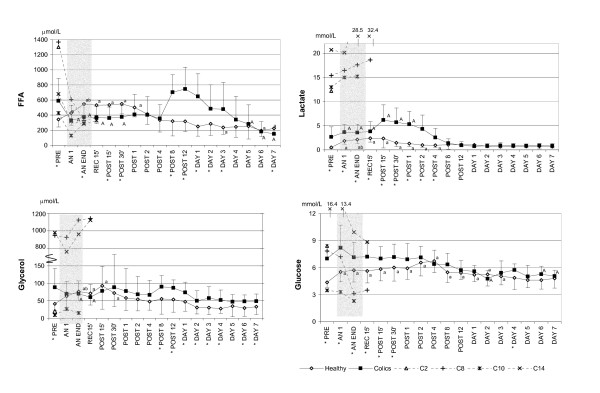
**Changes in plasma free fatty acids, glycerol, lactate and glucose in healthy and colic horses**. The mean values (SD) are shown from before anaesthesia to 7 days post anaesthesia in healthy (n = 9–20) and colic horses (n = 8–16) with the American Society of Anaesthesiologists physical status 5 (ASA 5) colic horses shown individually. Note that the time scale is not linear and that the Y-axis is broken for glycerol. FFA = plasma free fatty acids. PRE = before anaesthesia; AN 1 = after one hour of anaesthesia; AN END = end of anaesthesia; REC 15' = 15 minutes after discontinuation of inhalation anaesthesia, still recumbent; POST 15' and 30' = 15 and 30 minutes after recovery to standing; POST 1, 2, 4, 8, 12 = hours after standing; DAY 1, 2, 3, 4, 5, 6, 7 = days after anaesthesia. * Significant difference (p < 0.05) between groups. ^A ^(colics)^a ^(healthy) = significantly different from PRE. ^B ^(colics)^b ^(healthy) = significantly different from AN 1.

**Figure 3:**
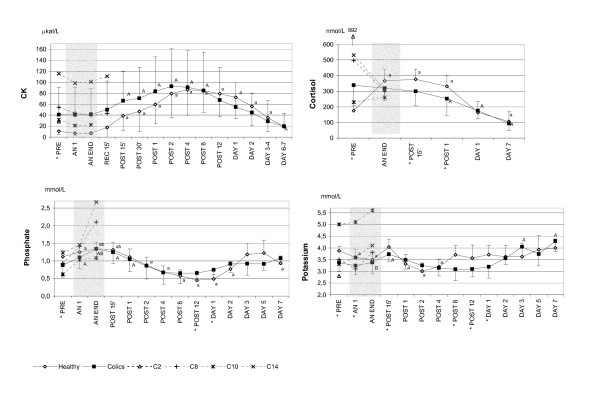
**Changes in plasma creatine kinase, cortisol, serum inorganic phosphate and potassium. **The mean values (SD) are shown from before anaesthesia to 7 days post anaesthesia in healthy (n = 8–20) and colic horses (n = 10–16) with the American Society of Anaesthesiologists physical status 5 (ASA 5) colic horses shown individually. Note that the time scale is not linear. CK = plasma creatine kinase; AN 1 = after one hour of anaesthesia; AN END = end of anaesthesia; REC 15' = 15 minutes after discontinuation of inhalation anaesthesia, still recumbent; POST 15' and 30' = 15 and 30 minutes after recovery to standing; POST 1, 2, 4, 8, 12 = hours after standing; DAY 1, 2, 3, 4, 5, 6, 7 = days after anaesthesia. * Significant difference (p < 0.05) between groups. ^A ^(colics)^a ^(healthy) = significantly different from PRE. ^B ^(colics)^b ^(healthy) = significantly different from AN 1.

In the study period from PRE to DAY 7, differences between groups and over time (interaction effect) were seen in serum albumin, protein, inorganic phosphate, potassium, total calcium, and chloride concentrations and plasma cortisol, glucose, glycerol, FFA, lactate, (Figs. 1, 2, 3 and Table 3). Within-group but not between-group differences were noted in haematology, plasma CK, and serum sodium levels (Figs. 1 and 3 and Table 3).

**Table 3:** The concentrations of serum total calcium, chloride and sodium in healthy and colic horses. The mean (± SD) concentrations of serum calcium (S-Ca), chloride (S-Cl) and sodium (S-Na) are shown from before anaesthesia to 7 days after anaesthesia with the American Society of Anaesthesiologists physical status 5 (ASA 5) colic horses shown individually. The figures within parenthesis are the number of horses included at each measurement.

	S-Ca			S-Cl			S-Na		
	*(mmol/L^1^)*			*(mmol/L^1^)*			*(mmol/L^1^)*		

	Healthy	**Colic**	*ASA 5*	Healthy	**Colic**	*ASA 5*	Healthy	**Colic**	*ASA 5*

PRE	2.9 ± 0.1(8)	2.6* ± 0.2 (16)	*2.4 ± 0.3 (4)*	92 ± 4 (8)	**93 ± 6 (16)**	*89 ± 7 (4)*	140 ± 3 (8)	**139 ± 4 (16)**	*136 ± 7 (4)*
AN 1	2.6^a ^± 0.1 (20)	**2.4^A ^± 0.2 (16)**	*2.1 ± 0.2 (3)*	91 ± 3 (20)	**94 ± 5 (16)**	*92 ± 5 (3)*	138 ± 4 (20)	**140 ± 4 (16)**	*140 ± 1 (3)*
AN END	2.6^a ^± 0.2 (20)	**2.3^A ^± 0.2 (16)**	*2.1 ± 0.0 (2)*	91 ± 4 (20)	**94 ± 5 (16)**	*91 ± 3 (3)*	137 ± 3 (20)	**138 ± 4 (16)**	*139 ± 2 (3)*
POST 15'	2.5^a ^± 0.1 (20)	2.3*^A ^± 0.2 (14)		90 ± 5 (20)	**92 ± 9 (14)**		135^a ^± 5 (19)	**135 ± 8 (13)**	
POST 1	2.5^a ^± 0.1 (19)	2.3*^A ^± 0.2 (13)		92 ± 4 (18)	**94 ± 5 (15)**		136 ± 4 (18)	**137 ± 4 (15)**	
POST 2	2.6^a ^± 0.2 (20)	2.3*^A ^± 0.2 (15)							
POST 4	2.8 ± 0.3 (19)	2.3*^A ^± 0.2 (15)		97 ± 6 (19)	**95 ± 5 (14)**		139 ± 5 (19)	**139 ± 5 (15)**	
POST 12	3.0 ± 0.2 (20)	2.3*^A ^± 0.2 (14)							
DAY 1	3.0 ± 0.2 (19)	2.4*^A ^± 0.2 (13)		99^a ^± 3 (19)	96* ± 4 (14)		139 ± 4 (19)	**138 ± 3 (14)**	
DAY 3	3.0^a ^± 0.1 (18)	2.8*^A ^± 0.2 (13)							
DAY 7	3.1^a ^± 0.1 (20)	3.0*^A ^± 0.1 (10)		98^a ^± 3 (20)	**96 ± 2 (10)**		139 ± 2 (20)	**137 ± 2 (10)**	

PRE = before anaesthesia; AN 1 = after one hour of anaesthesia; AN END = end of anaesthesia; REC 15' = 15 minutes after discontinuation of inhalation anaesthesia, still recumbent; POST 15' = 15 minutes after recovery to standing; POST 1, 2, 4, 12 = hours after standing; DAY 1, 3, 7 = days after anaesthesia.

* Significant difference (p < 0.05) between groups. ^A ^(colics)^a ^(healthy) = significantly different from PRE.

The highest lactate concentration in the post period was 12.4 mmol/L and was seen at POST 15' in the Shetland pony (C1). This individual also had the highest lactate PRE of the surviving horses (6.5 mmol/L).

### Muscle sample chemistry

ATP was lower and creatine and lactate were higher in colic horses at START (p = 0.036; p = 0.005; p = 0.0002) and END (p = 0.001; p = 0.017; p = 0.0005) of anaesthesia compared to healthy horses (Table 4). The lowest ATP content (13.9 mmol/kg d.w.) was found at END in a colic horse (C14). In the healthy horses ATP was decreased and IMP was increased at POST 1 (p < 0.008; p < 0.014) and DAY 1 (p < 0.01; p < 0.014) compared to END. Muscle lactate increased in both groups during anaesthesia (p < 0.0075; p < 0.001 in colic and healthy horses respectively). In one healthy horse (H14), lactate at END of anaesthesia had increased to a level similar to that in the ASA 5 horses (92.6 mmol/kg d.w.). This horse developed a post anaesthetic triceps myopathy. As it was an extreme outlier, this value is not included in Table 4.

**Table 4:** Gluteal muscle content of metabolites in healthy and colic horses. The muscle content of adenosine triphosphate (ATP), adenosine diphosphate (ADP), adenosine monophosphate (AMP), inosine monophosphate (IMP), creatine phosphate (CP), creatine (Cr) and lactate (La) from after induction of anaesthesia to one day after anaesthesia in healthy (plain style) and colic horses (bold) with the American Society of Anaesthesiologists physical status 5 (ASA 5) colic horses shown separately (italic). The figures within parenthesis are the number (n) of horses included at each measurement.

	ANAESTHESIA START			ANAESTHESIA END			POST 1			DAY 1		
*mmol/kg d.w*.												

	Median	Range	(n)	Median	Range	(n)	Median	Range	(n)	Median	Range	(n)

ATP	26.0	22.2–28.4	(20)	26.1	21.7–29.4	(20)	25.0^c^	17.3–27.3	(7)	22.4^c^	19.8–27.5	(6)
	23.0*	**17.9–32.6**	**(15)**	22.0*	**17.5–29.9**	**(14)**	**23.8**	**21.2–25.6**	**(7)**	**22.7**	**18.8–30.1**	**(13)**
	*24.4*	*16.4–25.1*	*(3)*		*13.9–24.3*	*(2)*						
ADP	4.0	3.4–4.1	(7)	4.3	3.7–4.4	(7)	4.0	3.2–4.2	(7)	4.0	3.7–5.0	(6)
	**4.3**	**3.7–5.2**	**(15)**	**4.5**	**3.6–5.3**	**(14)**	**4.5**	**4.0–5.2**	**(7)**	**4.3**	**3.9–5.2**	**(13)**
	*4.3*	*3.5–4.5*	*(3)*		*3.3–5.2*	*(2)*						
AMP	0.16	0.06–0.31	(20)	0.18	0.06–0.37	(20)	0.26	0.14–0.32	(7)	0.34	0.23–0.49	(6)
	0.28*	**0.20–0.35**	**(15)**	0.28*	**0.18–0.38**	**(14)**	**0.31**	**0.21–0.39**	**(7)**	**0.26**	**0.20–0.45**	**(13)**
	*0.24*	*0.16–0.25*	*(3)*		*0.23–0.35*	*(2)*						
IMP	0.11	0.00–0.57	(20)	0.06	0.00–0.43	(20)	0.17^bc^	0.00–0.35	(7)	0.24^bc^	0.07–1.04	(6)
	0.03*	**0.00–0.18**	**(15)**	**0.08**^B^	**0.00–0.41**	**(14)**	**0.07**	**0.03–0.12**	**(7)**	**0.05**	**0.00–0.15**	**(13)**
	*0.04*	*0.00–0.23*	*(3)*		*0.07–1.02*	*(2)*						
CP	60.4	41.5–89.2	(20)	65.2	32.3–83.0	(20)	58.9	50.6–75.3	(7)	54.6	43.4–68.0	(6)
	**56.1**	**26.7–77.0**	**(15)**	**44.1**	**19.7–77.8**	**(14)**	**53.0**	**31.3–73.5**	**(7)**	**65.8**^E^	**34.2–81.1**	**(13)**
	*70.7*	*22.8–74.6*	*(3)*		*60.3–62.5*	*(2)*						
Cr	55.8	52.2–67.1	(7)	59.8	56.5–74.4	(7)	53.0^bc^	44.0–56.0	(7)	47.4	39.1–80.5	(6)
	74.6*	**50.2–106.3**	**(14)**	82.1*	**49.5–111.9**	**(13)**	83.5*	**60.7–114.0**	**(7)**	66.4*^E^	**43.1–110.4**	**(13)**
	*63.5*	*54.2–109.0*	*(3)*		*68.2–129.9*							
La	13.8	5.9–29.5	(18)	20.0^b^	10.9–46.2	(19)	22.1^b^	6.1–34.0	(6)	16.5^ce^	10.4–39.1	(6)
	22.5*	**14.9–36.2**	**(15)**	31.3*^B^	**20.0–54.9**	**(14)**	**17.6**	**11.7–58.9**	**(7)**	**17.1**^C^	**8.9–37.5**	**(12)**
	*38.1*	*37.9–57.5*	*(3)*		*83.0–100.2*	*(2)*						

ANAESTHESIA START = shortly after induction of anaesthesia; ANAESTHESIA END = immediately before discontinuation of inhalation anaesthesia; POST = 1 hour after regaining the standing position after anaesthesia; DAY 1 = one day after anaesthesia;^B ^(colics)^b ^(healthy) = significantly different (p < 0.05) from START;^C ^(colics) or ^c ^(healthy) = significantly different from END; ^E ^or ^e ^= significantly different from POST 1.

Lactate for one healthy horse (H14) at END is not presented in the Table as the value is an extreme outlier compared to those of the rest of the group; the horse developed a severe (triceps) myopathy.

### Weight

**Figure 4:**
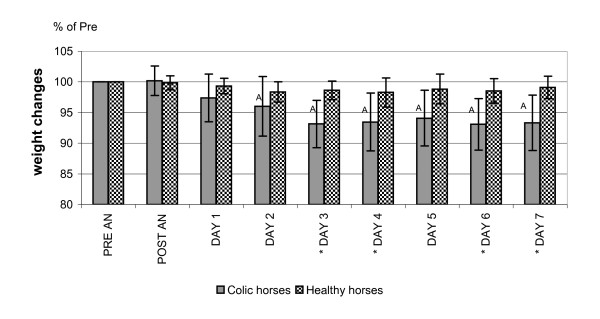
**Changes in weight over time in 7 healthy and 13 colic horses**. The mean weight changes (± SD) from before anaesthesia to 7 days post anaesthesia are shown as a percent of the weight pre-anaesthesia, which is regarded as 100%. PRE AN = before anaesthesia; POST AN = at removal from the recovery stall. DAY 1, 2, 3, 4, 5, 6, 7 = days after anaesthesia. * Significant difference (p < 0.05) between groups.

There was no significant difference in weight between groups at PRE (527 ± 106 kg in colic horses; 472 ± 35 kg in healthy horses). Individual weights are shown in Tables 1 and 2. Changes after anaesthesia were calculated as percentage of the weight at PRE, which was regarded as 100% (Figure 4). At a maximum, the colic horses lost a mean of 8% of their PRE weight. The horse that lost most weight (13%) was a Shetland pony (C1).

## Discussion

The results from analyses of blood parameters and muscle biopsy samples before, during and up to one week post anaesthesia show that metabolism pre- and post-anaesthesia differs between healthy horses and horses subjected to emergency abdominal surgery. The higher pre-anaesthetic levels of plasma cortisol, FFA, glycerol, glucose and plasma and muscle lactate at START in colic horses suggest a greatly increased sympathetic output, which profoundly affected the metabolic processes with activation of both the carbohydrate and lipid metabolic pathways [27,28].

### Physiological values in the preoperative period and during anaesthesia

Although probably underestimated on account of administered drugs, HR was higher in colic horses before anaesthesia than in healthy horses and was generally higher in the horses with the poorest prognosis. These findings are in accordance with earlier reports [4,5,8-10]. The increased HR in colic horses may be explained by pain and endotoxemia but also by hypovolemia in some cases [29].

During anaesthesia many colic horses experienced periods of severe hypotension, and acid-base and blood-gas disturbances. The low pH in many colic horses during anaesthesia was due to a mixed respiratory and metabolic acidosis. In most horses the acid-base balance was normalized at POST 1. Instituting mechanical ventilation in these patients was not always a possible treatment to hypercapnia, since IPPV tended to further impair pulmonary gas exchange, possibly as a result of further impaired pulmonary circulation [30,31]. Dobutamine therapy was associated in some cases with the development of cardiac dysrhythmia and was therefore used with caution.

### Metabolism before anaesthesia

The results of blood parameters in the colic horses in the present study confirm those of other studies, with increased circulating levels of lactate, glucose, FFA and CK [8,9,12,32]. Preoperatively the colicky horse experiences several stressors that may influence metabolism. Further, impaired tissue oxygenation due to depressed circulation may lead to lactacidaemia through anaerobic metabolism. In the critically ill human patient accelerated glycolysis produce excess amounts of pyruvate that not only enter the Krebs cycle but also form lactate [28,33,34]. At the same time as glycolysis is activated, there is also activation of lipolysis. Thus, the generalised stress response leads to parallel increases in plasma glucose, free fatty acids, triglycerides and lactate [28]. These findings in humans are well compatible with the observations in the present study. That anaerobic metabolism in muscle contributed to the lactacidaemia at PRE in the colic horses in the present study was supported by the observation that many colic horses and especially the ASA 5 horses had a low muscle content of ATP parallel with high muscle lactate at START. Low ATP and CP together with high creatine and lactate concentrations in muscle have also been found in the severely injured or septic human patient [35]. Some colic horses showed extremely high plasma lactate concentrations before surgery, the most severely ill horses usually having the most severe changes. Four horses (C2, 8, 10 and 14) out of the 9 colic horses that did not survive had, apart from severe clinical symptoms, PRE plasma lactate concentrations well above 10 mmol/L, which in several earlier studies have been found to be associated with a very poor prognosis of survival [5,8,12]. The decision regarding euthanasia instead of attempted surgery is not always easy to make when it is primarily based on the clinical impression of the patient. Lactate has repeatedly been reported to be a good predictor for survival and with the many new bedside analysers that have entered the market; lactate assay should no longer be more difficult to perform than blood gas analysis.

The finding of lower concentrations of serum potassium in the colic than in the healthy horses at PRE is in agreement with results in a recent study [36]. The low potassium levels in the colic horses may result from starvation due to their disease [37]. The healthy horses however were fasted for 12 hours before anaesthesia and this is comparable with the median duration of starvation of 13 hours in the colic horses. In human studies, more than half of randomly affected trauma patients present with low potassium levels. Further, the degree of hypokalemia has been shown to be associated with the severity of trauma and subsequent mortality in humans [38,39]. The low potassium level was explained by the stimulating effect of epinephrine or other beta-2 agonists on potassium uptake into muscle [33,39]. Similar mechanisms may possibly have influenced serum potassium concentrations in the colic horses in the present study.

### Metabolism in the anaesthetic period

In the present study it is not possible to differentiate between the effects of anaesthesia alone and of anaesthesia plus surgery in the colic horses. However, in some instances the changes were similar in both groups suggesting that anaesthesia was the major influencing factor.

The differences between the groups in FFA, glycerol and cortisol concentrations indicate that after induction of anaesthesia the sympathetic output decreased in the colic horses but increased in the healthy horses [27]. In previous studies, inhalation anaesthesia has been shown to induce a stress response with increases in adrenocorticotropic hormone and cortisol in healthy horses [40,41], a finding which is confirmed by the results of this study. The increased concentration of glucose during and after anaesthesia in the healthy horses in the present study may be an effect of the increased concentration of cortisol since this hormone has anti-insulin properties [42]. The increase in FFA and glycerol as seen during anaesthesia in the healthy horses in the present study is not a common finding [40,43]. It is possible that the duration of anaesthesia had an effect on FFA release since the increase was not significant until after three or four hours of anaesthesia and the duration of anaesthesia in the previous studies were approximately two hours. There is also a possibility that dobutamine which was infused in some healthy horses during anaesthesia may have been partly responsible for the significant increase in FFA. Lipolysis in horses is mediated by both β1 and β2-adrenoceptors [27], and dobutamine has an effect on both these receptors [44]. The dobutamine infusion rate in the healthy horses never exceeded 2.0 μg/kg/min and may be regarded as rather low, but is within the recommended range [45,46]. However, there were no differences in the FFA concentration changes between colic horses receiving dobutamine or not.

An interesting finding was that lactate in muscle increased in both colic horses and healthy horses from START to END of anaesthesia. This increase was not paralleled by similar increases in plasma, which, apart from an increased production due to hypoxaemia, may be due to decreased venous drainage causing an accumulation of produced lactate within the muscle during anaesthesia.

An increase in serum phosphate after induction of anaesthesia as seen in both groups in the present study has been reported earlier [47-49]. Johnson et al. [47] and Lindsay et al. [48] speculated that this phosphate derived from dephosphorylation of CP and ATP, since these were the most likely sources of phosphate. No significant changes in CP or ATP from START to END of anaesthesia were observed in either colic or healthy horses in the present study. Serum phosphate concentrations may be affected by acidosis, but although most of the colic horses in the present study were acidaemic there were no differences in phosphate between the groups during anaesthesia. Watson (2002) suggested that the increase in phosphate occurring together with a decrease in calcium implied an alteration in renal function. A calcium decrease in serum was seen in both groups during anaesthesia in the present study and may have been a result of the increased serum phosphate [50]. Renal causes therefore cannot be excluded.

Notable was large inter-individual variation in the stores of muscle phosphagen in the colic horses likely related to the severity of disease and circulatory compromise. The increased content of creatine in the muscle in colic horses may be due to an increased breakdown of CP [51]. There were no statistically significant differences in CP between the groups, but there was a trend towards lower values in colic horses indicating a greater demand for anaerobic metabolism. In an earlier study [16] we found a decrease in muscle CP at the end of 2.5 hours of propofol-ketamine anaesthesia in healthy horses, which implies that during anaesthesia there is a demand for energy through anaerobic metabolism even in the healthy horse.

### Metabolism in the post-anaesthetic period

The increase in plasma lactate seen after recovery to standing in the colic horses could be related both to a phenomenon of lactate wash-out when tissue perfusion increases after standing [19,20], and there may also have been a slight increase in production of lactate during the work of standing. Lactate within the muscle cell is rapidly removed or consumed when circulation is restored [52], which may explain why lactate in the muscle sample taken 1 hour after standing had decreased or remained at anaesthetic levels.

There were no differences between the healthy and colic horses in muscle lactate at POST 1 and in plasma lactate and glucose from POST 8 onwards, indicating normalization of glycolysis and tissue perfusion. Instead, FFA increased abruptly in the colic horses at POST 8 and reached a maximum level at POST 12. Simultaneously a smaller but significant increase was noted in glycerol. Similar changes have been reported previously in the post-operative colic horse [13]. In this period, colic horses have an altered or increased basal energy demand compared to healthy horses as a result of the surgical intervention and recovery from illness [53,54]. Concomitantly, little or no new energy through feed is usually provided and some of the energy reserves of the colic horses may already have been consumed before anaesthesia. Several studies have shown an increase in FFA and glycerol levels after fasting, reflecting an increased fat mobilization and a breakdown of triglycerides [55,56]. There was considerable individual variation among the colic horses in the rate at which FFA decreased. In some horses low levels comparable to those in healthy horses were reached on DAY 2, while in others this was not seen until DAY 7. This discrepancy probably reflects variations in feed intake and differences in the energy demand after the surgical intervention.

The decreases in serum potassium in the post-anaesthetic period in both groups may also partly depend on the decreased intake of feed [37], at least in the colic horses, but again, the hormonal effect on potassium uptake into muscle can not be disregarded [33].

Also notable in the post-anaesthetic period was the hypophosphatemia that occurred in both groups. The healthy horses even showed a decrease to a slightly but significantly lower level than the colic horses (at POST 12). Since the same pattern of changes in phosphate was seen in both groups, the fluctuations would seem to be more closely related to the anaesthetic procedure and the recovery phase than to surgery or illness. A post-anaesthetic decline in inorganic phosphate has been reported in tables from earlier studies of healthy horses, but little attention has been paid to this finding [48,57]. Acute hypophosphatemia may have several causes such as sepsis, diarrhoea, and glucose infusion, while renal tubule disorders may lead to hypophosphatemia on a more long-term basis [58]. In most horses in the present study that showed hypophosphatemia none of the above-mentioned acute disorders was present. Reinstitution of feeding in starved people and horses is associated with hypophosphatemia [59,60], but in such cases the phosphate decrease has a slower onset than was seen in the present study.

An increased urinary excretion of inorganic phosphate after abdominal surgery has been described previously [36], however, neither renal function nor urinary phosphate were assessed in the present study. It is of interest to note that in humans, rhabdomyolysis is one of the most common and reproducible consequences of hypophosphatemia [61,62]. The changes in phosphate in the horses of the present study were rather short lasting, but may be a factor to consider in the development of postoperative myopathies occurring after recovery to standing. Interestingly, in a case report on two horses that developed general myopathy after anaesthesia and were subsequently euthanised, a severe hypophosphatemia and hypokalemia was observed [63]. Phosphate is important for cellular metabolism, and if not available, this will influence both the ATP, CP and 2,3 diphosphoglycerate (2,3-DPG) stores [64]. Limitation in the stores of ATP and CP can lead to a lower energy release, and a limitation in 2,3-DPG can lead to diminished oxygen release to the cells and thus cellular dysfunction [64]. In addition, phosphate is needed for the phospholipids, an important part of the cellular membranes.

Whereas the changes in phosphate both during and after anaesthesia were similar in healthy and colic horses, the changes in total calcium differed between groups post anaesthesia, with a prolonged decline in the colic horses. A similar decrease in calcium was found postoperatively in colic horses of an earlier study [36]. Whereas no correlation with albumin was found in that study, calcium changes in the present study followed the changes in albumin both during and after anaesthesia. Since approximately 50% of total calcium is bound to protein, changes in albumin may account for the observed changes in calcium during the study period [65]. One hypothesis for membrane disturbances and cell injury is that an influx of calcium will activate calcium-dependent enzymes, leading to tissue damage [66].

The decreased content of ATP in the healthy horses at DAY 1 compared to END supports an earlier study where we found decreased contents of both ATP and CP the day after anaesthesia in healthy horses [16]. An increased demand for ATP may have been expected immediately in response to the work of standing after anaesthesia. Under normal circumstances the regeneration of ATP is fast and has been reported to have returned to baseline levels within 15 minutes of a maximal treadmill exercise [67,68]. By one hour after regaining the standing position, ATP should have been regenerated given there was no obvious limitation of either oxygen or substrate. At least in the healthy horses, there is no reason to believe that either oxygen or substrate would be a limiting factor. One could speculate that a decreased capacity for regeneration of ATP may have developed due to the metabolic and electrolyte changes that occurred during and post anaesthesia. The rise in CK in the POST period also indicates a disturbed membrane function [66].

The results of the present study clearly indicate that marked metabolic disturbances remained in the colic horses several days after anaesthesia. Even in the healthy horses metabolism was affected as shown for example by the decreased content of ATP at POST 1 and DAY 1.

### Complications and outcome in the postoperative period

Of the four horses that were euthanised after having recovered from anaesthesia, three developed severe complications, which were the direct reasons for euthanasia (Table 2). Some metabolic changes occurred during the last hours before euthanasia in these horses that differed from the changes in the surviving horses. Plasma lactate for example began to increase in two horses (C11 and C19) during the last hours before euthanasia, indicating a deteriorating condition. Very high as well as very low concentrations of glycerol and FFA were observed emphasising the complex nature of the metabolic response to disease and therapy. Although post-anaesthetic complications were also seen in the group of healthy horses, analytes of the healthy horses that developed complications diverged little from those from the rest of the healthy horses.

Despite the fact that colic horses entered anaesthesia with higher concentrations of CK than healthy horses, increases to similar concentrations in the two groups were seen after anaesthesia. In line with some human studies [69], however, no clear association, was found between the degree of gait disturbances and the concentration of CK post anaesthesia in either group of horses. Some of the horses with the highest CK values did not show any gait dysfunction, at least at walk.

The concentrations of plasma FFA, lactate and cortisol at PRE and during anaesthesia seemed to have more influence than the quality of recovery on the POST levels. This explains the absence of a clear association between the quality of recovery and the POST 15' or POST 30' concentrations of these analytes in the colic horses. In the healthy horses there appeared to be a somewhat closer relationship.

The high incidence of post-anaesthetic complications in the healthy group of horses (four horses with gait dysfunction, two of which showed clinical symptoms of myopathy) may be explained by the prolonged duration of anaesthesia of nearly four hours. The incidence of post-anaesthetic complications has been shown to increase as the duration of anaesthesia increases [3,70,71].

Some values of measured parameters from these horses diverged from the rest of the other healthy horses but had little effect on the calculated mean values why the results from the healthy group of horses still may be considered representative for a healthy group of horses anaesthetised with the present protocol.

The limited number of horses included in the study and the variable horse material in the colic group decrease the power of the study. A statistician was consulted and selected an appropriate statistical method for the present material. Most results are also in accordance with earlier presented studies [5,9,12,13,32].

### Post-anaesthetic weight changes

The mean weight loss in our colic horses is similar to that in earlier studies [15,36]. Remarkably, two colic horses did not lose weight at all despite that they both suffered from serious complications postoperatively. The healthy horses also showed a tendency, although not significant, to a negative weight balance during the week following anaesthesia. The weight loss and the increased lipid metabolism in the postoperative period indicate an ongoing breakdown of body reserves that would be desirable to reduce. Some studies have addressed parenteral nutrition in the post-surgical equine colic patient [14,72]. It would be interesting also to study protein metabolism and amino acid turnover in colic horses postoperatively.

### Influence of treatment and induction protocol

Since some colic horses had spent some time at the clinic by the time the PRE sample was taken, this sample may have been influenced by the volume of administered fluids and the analgesic substances provided to the horse. In five horses, a sample obtained on arrival (not reported here) indicated that different degrees of hemodilution (decreases in Hb, Hct, serum protein and albumin) had occurred before the PRE sample. Lactate, glucose and cortisol do not show equivalent decreases but, rather, increases from arrival to PRE, and most likely some differences between colic horses and healthy horses are obtunded or underestimated as a result of hemodilution. As judged by the similar changes in total protein and albumin, hemodilution occurred in both colic and healthy horses during anaesthesia [73]. Sequestration by protein in the peritoneal cavity as well as intestinal losses [9,74] may also have played a significant role when low protein and albumin concentrations were observed at the same time as normal to high Hb and Hct were measured. Total protein did not differ between the groups from DAY 2, but it was not until DAY 7 that albumin was similar in the two groups. Increases in inflammatory proteins may be accounted for the earlier rise in total protein in the colic horses [13,75]. The rate of albumin synthesis has been found to be reduced after abdominal surgery in man [76], and this factor may therefore also need to be considered.

Three different induction protocols were used in the colic horses although the majority (15 horses) were induced with thiopentone and guaifenesin. There is a possibility that this may have influenced the results. However, after one hour of anaesthesia most effects of the induction protocol were likely overridden by the effects of the inhalant [77].

## Conclusion

The surgical colic cases displayed different degrees of metabolic stress with parallel activation of lipolysis and glycolysis and a depletion of muscle energy reserves before anaesthesia. The horses with the most severe pre-anaesthetic metabolic derangements died or were euthanised during anaesthesia or in the recovery box. Anaesthesia and surgery relieved the colic horses from some of the general stress response whereas anaesthesia induced a stress response in the healthy horses. Muscle oxygenation was insufficient during anaesthesia in both groups, with increases in lactate in muscle and plasma, although the levels were higher in the colic horses. The post-anaesthetic period was associated with increased lipolysis and weight loss in the colic horses, indicating a negative energy balance at least during the first week post-operatively.

## Sammanfattning

Att metabolismen är förändrad hos kolikhästen jämfört med den friska hästen är välkänt. De uppvisar ofta förhöjda blodnivåer av bland annat laktat, glukos, fria fettsyror och cortisol vilket tyder på ett stresstillstånd med aktiverad kolhydrat-och fettomsättning samt nedsatt perifer cirkulation. Kolikhästar har markant förhöjd mortalitet i samband med anestesi jämfört med den friska hästen och flera studier har påvisat ett samband mellan laktatnivån i plasma och sannolikheten att dö i samband med anestesi. Det saknas dock studier som närmare studerat metabolismen i hästens största energireservoar, nämligen skelettmuskeln.

Denna studie beskriver de metabola förändringarna i blod och muskel före, under och upp till en vecka efter anestesi hos 20 kolikhästar som buköppnats akut samt hos 20 friska hästar som bara sövts och placerats i ryggläge men utan kirurgiskt ingrepp. Muskelmetabolismen har studerats genom analys av ATP, ADP, AMP, IMP, CP och laktat i biopsier från gluteusmuskeln. I blod och plasma har markörer för stress, kolhydrat-respektive fettomsättningen, elektrolyter, muskelenzymer samt hematologiska parametrar studerats.

Före anestesi hade kolikhästarna signifikant högre nivåer i plasma av laktat, glycerol, glukos, fria fettsyror, cortisol och CK medan serumkalium och fosfat var lägre än hos de friska hästarna. I muskeln sågs förhöjda nivåer av laktat medan ATP nivån var låg hos flera kolikhästar. Under anestesi sjönk plasmakoncentrationen av fria fettsyror och glycerol hos kolikhästarna medan koncentrationerna ökade hos de friska hästarna. Laktat i både muskel och plasma ökade i båda grupperna under anestesi och ökade ytterligare i plasma hos kolikhästarna efter resning. Inom ett dygn efter resning steg fria fettsyror samt glycerol i plasma hos kolikhästarna varefter en successiv sänkning observerades. En markant sänkning i serum fosfat under det första dygnet efter anestesi sågs hos båda grupperna medan CK ökade. En vecka efter anestesin var de flesta parametrar på samma nivåer i båda grupperna. Kolikhästarna förlorade i genomsnitt 8% av sin ursprungliga vikt. Elva kolikhästar överlevde minst en vecka efter anestesin.

Sammanfattningsvis sågs en ökad kolhydrat-och fettomsättning hos kolikhästarna upp till en vecka efter anestesin vilket kan förklara viktminskningen i återhämtningsfasen. De ökade laktatnivåerna i plasma såväl som i muskel tillsammans med minskade nivåer av ATP och CP i muskulaturen hos flera kolikhästar tyder på en ökad anaerob metabolism. De låga fosfatnivåerna efter anestesi är anmärkningsvärda särskilt som hypofosfatemi hos människa anges som en viktig faktor för utvecklandet av myosit.

## Competing interests

The author(s) declare that they have no competing interests.

## Authors' contributions

AE planned the study, carried out the practical work (collecting samples and data) as well as prepared the major part of the manuscript.

GN and BEG participated in designing the study, interpretation of the results and in critically revising the manuscript.

All authors read and approved the final manuscript.
